# 

**Published:** 1914-11-14

**Authors:** 


### CONTENTS.

PAGE

**The Drug Treatment of Disease** ... ... ... 141

**The Fees of Institution Medical Officers** ... 142

**Hospital and Institutional News** ... ... ... 143

Boots for the Belgian Wounded—The North Midland Clearing Hospital Leaves for the Front—The Boot Difficulty at Gravesend—The Salary of an Irish Hospital Secretary—Cardiff Scheme for Visits to Wounded—Accommodation for Mental Defectives—The Salary of Mental Hospital Superintendents—The Celebration of a Jubilee—Medical Men as Prisoners of War—The Leaven of Progress—Hospital Concerts for London Recruits — Glasgow Hospitals and Municipal Control—Poor-Law Infirmaries and the War—Radium in Poor-Law Infirmaries—Specialists for London Infirmaries—No Funds for Sanatorium Benefit—Prison Doctors and Active Service—The Influence of Religion on Mental Patients—A Guernsey Hospital President Resigns — A City Hospital for the Wounded—A Weak Point in the "Following-up" System — Final Convalescent Home Arrangements in Scotland—The New International Hospital at Tokyo—The Gate of English Hospitality—Death of Beau Nash's Successor at Bath—The "War Schedule" of Insurance Drugs—The London Ambulance Service—The Art of Appealing—This Week's Drug Market.

**The Royal Army Medical Corps and the War** 149

The Questions of Age and Maximum Efficiency.

**A "Close" Time for Emotional Givers** ... 150

**The Making of a Military Hospital** ... ... 151

How the Transformation at Cardiff was Effected.

**Hospitals and the War** ... ... ... ... 152

**Work for the Wounded of the Allied Armies**

St. George's Hospital—Barnsley: Cases from Sheffield—Bristol—Cardiff: Wounded Sent Direct from France—Leicester: The 5th

[*See over.*]

### CONTENTS—(*continued*).

PAGE

Northern General Hospital—Ancoats Hospital: Belgian Appetites — Middlesbrough—Sheffield: The Ambulance Problem—Wakefield—Worthing: First Batch from the Front—Ashton-under-Lyne: Anti-typhoid Vaccine—Bristol: 400 British and Belgian—St. Ives (Cambs) Red Cross Hospital.

**The Royal Infirmary, Glasgow** ... ... ... 155

I. The Genesis of the Scheme.

**National Insurance Act Developments** ... 157
A Voluntary Insurance Bill.

**Round the World** ... ... ... ... ... ... 159

Fellow-Workers and the Roll of Honour.

**The London Panel Committee** ... ... ... 160

**The Editor's Letter-Box** ... ... ... ... 162

State Children and the War.

PAGE

**Care for Nerve Cases** ... ... ... ... 160

**Medical Appointments** ... ... ... ... 162

**Buildings Contemplated** ... ... ... ... 162

**The Book Market** ... ... ... ... ... 162

**The Institutional Worker** ... ... (Suppl.) 1
Answers to October Questions.
Enquiries *re* Condition of Patients—Padded Rooms.
Questions for November.
Questions Invited.
Employment and Training.
War Matters.
The Sick and in Need.
Practical Hints.
Miscellaneous.
Acknowledgments.

